# Considerations and challenges for sex-aware drug repurposing

**DOI:** 10.1186/s13293-022-00420-8

**Published:** 2022-03-25

**Authors:** Jennifer L. Fisher, Emma F. Jones, Victoria L. Flanary, Avery S. Williams, Elizabeth J. Ramsey, Brittany N. Lasseigne

**Affiliations:** Department of Cell, Developmental and Integrative Biology, Heersink School of Medicine, University of Alabama at Birmingham, Birmingham, AL 35294 USA

**Keywords:** Sex differences, Drug repurposing, Sex-bias, Sex-aware, Review, Therapeutics, Pharmaceuticals, Computational drug repurposing

## Abstract

Sex differences are essential factors in disease etiology and manifestation in many diseases such as cardiovascular disease, cancer, and neurodegeneration [33]. The biological influence of sex differences (including genomic, epigenetic, hormonal, immunological, and metabolic differences between males and females) and the lack of biomedical studies considering sex differences in their study design has led to several policies. For example, the National Institute of Health’s (NIH) sex as a biological variable (SABV) and Sex and Gender Equity in Research (SAGER) policies to motivate researchers to consider sex differences [204]. However, drug repurposing, a promising alternative to traditional drug discovery by identifying novel uses for FDA-approved drugs, lacks sex-aware methods that can improve the identification of drugs that have sex-specific responses [7, 11, 14, 33]. Sex-aware drug repurposing methods either select drug candidates that are more efficacious in one sex or deprioritize drug candidates based on if they are predicted to cause a sex-bias adverse event (SBAE), unintended therapeutic effects that are more likely to occur in one sex. Computational drug repurposing methods are encouraging approaches to develop for sex-aware drug repurposing because they can prioritize sex-specific drug candidates or SBAEs at lower cost and time than traditional drug discovery. Sex-aware methods currently exist for clinical, genomic, and transcriptomic information [1, 7, 155]. They have not expanded to other data types, such as DNA variation, which has been beneficial in other drug repurposing methods that do not consider sex [114]. Additionally, some sex-aware methods suffer from poorer performance because a disproportionate number of male and female samples are available to train computational methods [7]. However, there is development potential for several different categories (i.e., data mining, ligand binding predictions, molecular associations, and networks). Low-dimensional representations of molecular association and network approaches are also especially promising candidates for future sex-aware drug repurposing methodologies because they reduce the multiple hypothesis testing burden and capture sex-specific variation better than the other methods [151, 159]. Here we review how sex influences drug response, the current state of drug repurposing including with respect to sex-bias drug response, and how model organism study design choices influence drug repurposing validation.

## Highlights

- Genetic, epigenetic, hormonal, immunological, metabolic, and environmental factors affect sex-biased drug responses.
- Drug repurposing approaches provide a significant advantage over novel drug development by reducing lengthy and costly clinical trials.
- Advances in compute processing power and optimized algorithms for computational systems have increased the efficiency and feasibility of computational drug repurposing.
- Multiple challenges still need to be addressed for sex-aware drug repurposing, including the insufficient understanding of the cause of variation of drug responses due to sex differences, better performing sex-aware repurposing methods, and the lack of large and balanced datasets to develop improved methods.
- Future low-dimensional representations of molecular association and network approaches could significantly impact the field of sex-aware drug repurposing.

## Background

### Introduction

Attempting to isolate novel therapeutic drug candidates can cost one to two billion dollars and 12–16 years of research [1]. As an alternative, drug repurposing strategies require less investment and lead to faster Food and Drug Administration (FDA) approval because repurposed candidates are already FDA-approved for alternative indications. Historically, drug repurposing has been serendipitous [1]. For example, hydroxychloroquine was initially approved only to treat malaria and later repurposed to treat other autoimmune diseases such as systemic lupus erythematosus (SLE). This repurposing resulted from retrospective clinical studies that found patients with SLE had better outcomes when treated with hydroxychloroquine for other conditions besides SLE [2]. Another fortuitous drug repurposing example, sildenafil, was initially intended for ischemic chest pain. However, after phase I clinical trials, it was repurposed to treat erectile dysfunction because of the unintended therapeutic effect reported [1]. Recently, through advancements in computational approaches, drug repurposing has become more systematic in predicting drug candidates that are effective and avoid adverse events [1]. This review will discuss the following drug repurposing categories and how they apply to sex-aware drug repurposing: data mining, ligand-target binding prediction, molecular associations, and network computational drug repurposing.

The effects of sex differences are known to lead to variation in therapeutic outcomes. For example, tumor resection followed by radiation and treatment with temozolomide is the standard treatment for Glioblastoma Multiforme (GBM) and is more efficacious in females [3]. This might be because females are more likely to have the DNA repair enzyme O6-methylguanine-DNA methyltransferase (MGMT) promoter methylated, a biomarker for a therapeutic response for temozolomide [4]. Another example of a sex-bias drug response is ibuprofen. This over-the-counter medication is more effective in males than females, even though no pharmacokinetic differences between the sexes have been identified [5]. However, pain receptors and nociception differences connected to estrogen activity in the nervous system might cause this variation in drug response [6]. Even though several examples of sex-bias drug responses exist, most drug repurposing methods do not consider sex. In addition, the available sex-aware drug repurposing methods require algorithmic improvements (e.g., potentially incorporating additional data types) to achieve better performance characteristics in order to improve sex-aware drug repurposing [7].

Adverse events, defined by the FDA as any undesirable experience using a medical product in a patient [8], are the fourth leading cause of death in the United States and can be caused by many factors such as tissue differences, age, development, and race [7, 9]. However, in 2001, eight of the ten drugs recalled by the FDA were more significant health risks to women than men [10]. This recall led to several studies that found that women are twice as likely to experience an adverse drug event compared to men based on adverse drug event case reports from the Food Drug Administration’s Adverse Event Reporting System (FAERS) or World Health Organization’s VigiBase database [7, 11, 12, 13]. Recently, during the coronavirus disease of 2019 (COVID-19) pandemic, there was an increase in the sex-bias adverse event (SBAEs) gap between females and males, possibly due to the pandemic exacerbating known SBAEs such as anxiety [9]. Even though SBAEs are more common in females, males are more likely to have a severe drug adverse event than females [13]. For instance, ranitidine (an antihistamine and antacid) causes duodenal damage in males [14]. Currently, there are several drug repurposing methods to identify drugs that might cause adverse events [15, 16]. Still, most methods that identify SBAEs, such as AwareDX, have significant limitations and would benefit from improvements to their accuracy [7, 11, 17–19].

To summarize, sex differences influence drug safety and efficacy, but drug repurposing, as a field, rarely considers sex differences when selecting or prioritizing drug candidates. In this review, we discuss biological mechanisms causing sex-dependent drug responses. In addition, we summarize current drug repurposing methods, survey cases where it has been done, and consider the challenges of developing and evaluating new drug repurposing candidates in light of sex.

## Main text

### Mechanisms driving sex-dependent variation in drug response

**Fig. 1:**
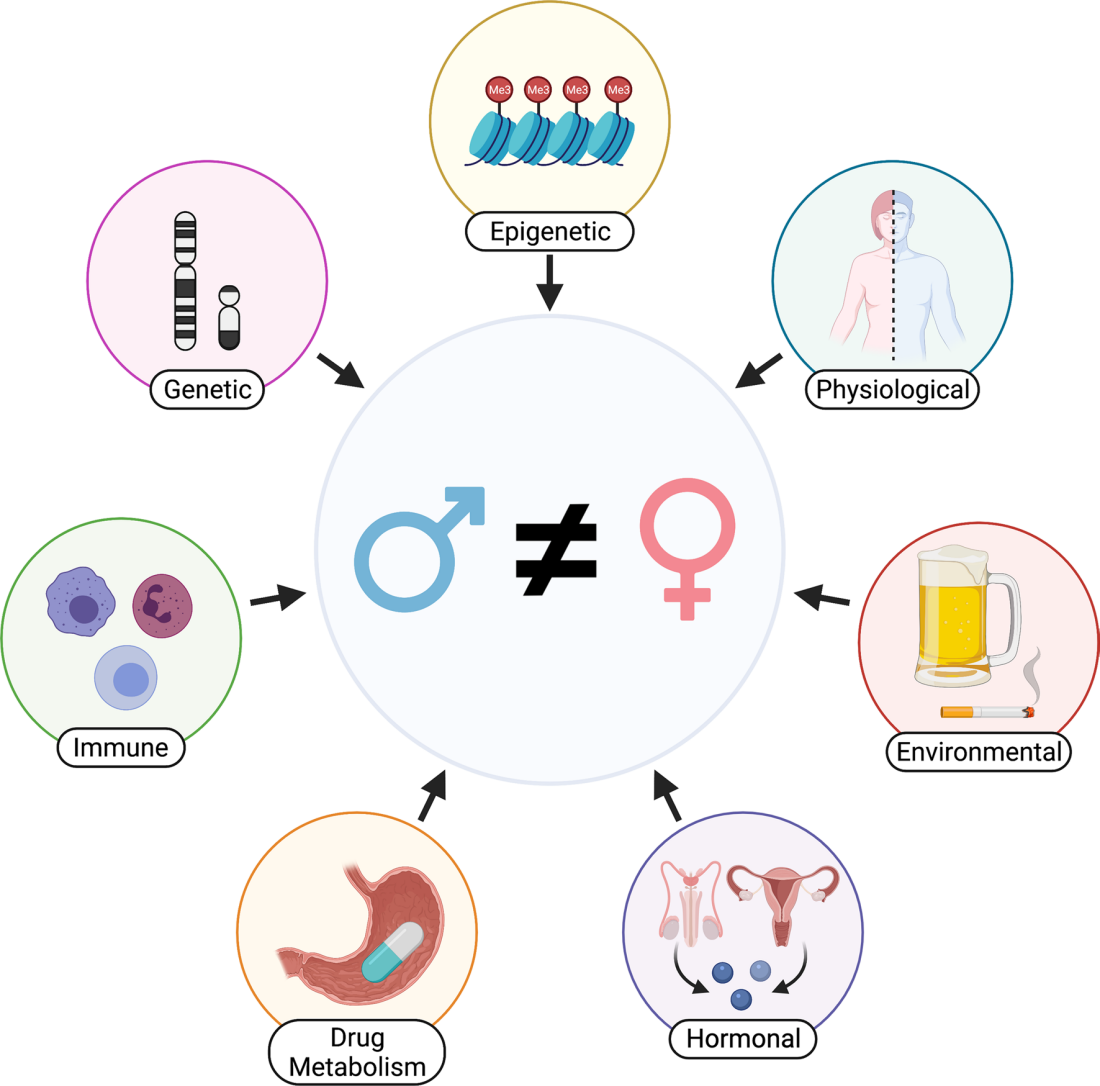
Factors known to influence sex-biased drug response include genetic, epigenetic, hormonal, immunological, metabolic, and environmental factors

In this review, we describe the current challenges and progress in the field of sex-aware drug repurposing by reviewing variations in drug response due to sex differences (Fig. 1). Here sex refers to the XX (female) or XY (male) genotype of an individual and is the focus of this review. Intersex and genotypes other than XX or XY, have not been extensively studied through future study is necessary and warranted. Gender refers to the societal construct of roles for women and men, which do not always overlap with biological sex [20]. Thus, we use sex to refer to a person’s genotype and gender to refer to a person’s social behavior. Sex differences can be sexually dimorphic, meaning a gene or phenotype is present in one sex but not the other, or sex-biased, meaning there are differences in effect or effect size between the sexes. In this review, we use sex differences and sex bias interchangeably.

Aside from environmental and social factors, almost all documented molecular sex differences arise from differences in the sex chromosomes, where mammalian females have two copies of the X chromosome and males have one X and one Y chromosome. This basic genetic difference leads to changes in gene expression that give way to larger-scale phenotypic changes as an organism continues to develop. During fetal development, the SRY gene on the Y chromosome codes for “maleness”, becoming especially apparent when dysfunction of the SRY gene leads to XY-genotyped individuals developing female sex characteristics [21]. However, sex-biased genes are not limited to sex chromosomes and can originate from autosomes [22]. When an organism has reached adulthood, many sex differences in gene expression are tissue-specific, with a large amount of differentially-expressed genes in tissues such as adipose, liver, and breast; but this can vary depending on the population of cell types in a given tissue [22, 23, 24]. In addition, multiple biological pathways have sex-biased gene expression and transcriptional regulation, including sex-biased expression quantitative trait loci (eQTLs) [22]. Sex-biased gene expression has been associated with sex-biased diseases, and they are more likely to be drug targets of FDA-approved drugs than non-sex-biased genes [25].

In addition to genetic differences, there are also epigenetic disparities between males and females, specifically in DNA methylation and histone acetylation and methylation [26, 27]. For example, DNA CpG island methylation is strongly associated with X-chromosome inactivation (XCI), which controls gene dosage compensation and has sex-specific patterns [28]. Lyonization (i.e., XCI) randomly inactivates either parental or maternal X chromosome copy resulting in tissue mosaicism, differential expression of parentally imprinted genes, and increased expression for genes that escape XCI [29]. About 15% of genes on the inactive X chromosome are consistently still expressed, and an additional 10% variably escape inactivation. This results in a ‘double dosage’, which leads to a higher level of gene expression in females [30, 31]. Tissue mosaicism and inheritance of both parental imprints of the X chromosome, as opposed to only the maternal imprint [32], may protect females from deleterious alleles [33]. For histone modifications, neonatal male and female mice brains have been found to be sexually dimorphic in histone H3 Lys9 acetylation (H3K9/14Ac) and trimethylation (H3K9me3) [27]. Additionally, there is documentation of sex-differentiated distribution of epigenetic marks such as histone H3 Lys27 trimethylation (H3K27me3), which is also associated with heterochromatic gene repression and X-chromosome inactivation [22]. Thus, these differences should be considered when identifying novel cancer drugs, many of which are epigenetic inhibitors that target DNA methyltransferases, histone deacetylases (HDACs), and histone methyltransferases [34]. Epigenetic modifiers have the potential to disrupt female dosage compensation, and the chemotherapy HDAC-inhibitor drug vorinostat has been shown to affect levels of H3K27me3 [34].

Many epigenetic changes that occur early in development are mediated by hormones. It is well established that males and females have differences in sex steroid hormones such as testosterone, estrogen, and progesterone [35]. These hormones vary in production site, blood concentration, and organ interactions [36]. For example, males produce testosterone in the testes and produce more testosterone than estrogen, while females produce more estrogen and progesterone. These hormones, with many others, are necessary for reproduction [37]. Sex hormones modulate body mass and fluids, enzyme synthesis, synthesis of triglycerides and high-density lipoprotein, and glucose metabolism [38], all of which can affect drug processing. Through steroid response elements [39] and G-coupled protein receptors [40] sex hormones affect gene expression, intracellular signaling, and downstream drug processing. Estrogen receptors, an example of steroid response elements, have an impact on energy intake and expenditure, regulation of adipose tissue distribution, insulin sensitivity, and the function of macrophages and immune cells [41]. These sex hormone signaling mechanisms may lead to downstream sex differences in endocytosis of drug transporters, therefore affecting drug response [19].

Sex chromosome genes and sex hormones, in addition to environmental and age-related factors, heavily influence immune responses [42]. Females have a higher antibody response, increased amounts of immunoglobulin, and a larger frequency of B cells than males, which leads to their ability to have a more robust immune response [42]. XCI may be one of the main influences of increased immune response in females because the inactive X chromosome can become reactivated in lymphocytes, resulting in the overexpression of autoimmune genes [43]. These sex differences in immune responses lead to differential susceptibility to autoimmune diseases, which disproportionately affect females [44, 45], and certain cancers, which disproportionately affect males [42]. The observed discrepancy in autoimmunity could, in part, be due to sex differences in the microbiome affecting sex hormone regulation [46]. These immune system inconsistencies may explain different pharmacokinetic responses to vaccines and various immunologic drugs [47].

There are many differences in all stages of pharmacokinetics between males and females including drug absorption, distribution, metabolism, and elimination [48]. Some of the contributing factors to differential drug absorption rates are variations in gastric enzymes, transporter proteins, and liver and kidney organ function [49]. For example, females have a higher gastric pH than men, which can increase the absorption of compounds such as caffeine through decreased ionization of weak bases [49]. In terms of the distribution of a drug throughout the body, plasma volume, body mass index (BMI), average organ blood flow, total body water versus body fat, and cardiac output all have sex differences [49]. Since females have a higher fat composition than males, the volume of distribution differs depending on whether a drug is lipid-soluble or water-soluble. In this scenario, a water-soluble drug would have a higher volume of distribution in males than in females, and vice versa for lipid-soluble drugs [48]. A higher volume of distribution results in higher concentration, so drug dosages should compensate for these effects to avoid the risk of adverse side effects. Pregnancy can cause changes in the elimination half-life of drugs, so the dosage requirements for drugs also need to be adjusted for pregnant individuals [50].

The drug zolpidem, also known as Ambien, is a drug the FDA recommended to be given to females at a dose half that of males; however, this recommendation only increased the adjusted dosage compliance from 10 to 15% [51]. The FDA zolpidem statement was due to pharmacokinetic and pharmacodynamic differences causing lower rates of clearance in women, resulting in 40–50% higher concentrations of the drug and a higher likelihood of side effects such as extreme drowsiness, possibly from non-compliance with the FDA dosage recommendations [52]. Zolpidem is among many drugs affected by the myriad of differences in drug metabolism in the cytochrome p450 (CYP) enzymes. Sex differences in the CYP superfamily of genes, which are involved with phase I drug metabolism, can explain some discrepancies in pharmacokinetic processes between males and females [53]. For example, differences in CYP genes such as CYP1A2, CYP2B6, CYP2E1, CYP3A4, affect the metabolism of hundreds of compounds [54]. CYP2B6 has more than 70 substrates (including ketamine), and females have higher overall activity compared to males [54]. CYP3A4 is involved in the metabolism of over 50% of all drugs (including zolpidem), and females have been found to have 20–50% higher activity than males [54]. In addition, the CYP superfamily of genes is involved in sex hormone biosynthesis [55].

Finally, differing environmental and social pressures can lead to conscious decisions that physically affect the body, resulting in differing responses to various drugs. These social pressures vary depending on gender, not just biological sex. Gendered behaviors may lead to changes in testosterone levels in men and women [56]. One example of environmental and social contributions is melanoma: men are more likely than women to develop melanoma and have a fatal outcome [57]. The increased likelihood of development could be due to many behavioral differences: men spend more time outside, are less likely to wear sunscreen, and are less likely to self-detect and examine for skin irregularities [58]. Males and females have different immune responses, so women’s higher melanoma survival rate could be due to women’s immune systems being more effective at preventing metastasis through estrogen signaling [57]. Women are also more likely to use supplements, natural botanicals, and homeopathic remedies, which are less likely to be reported than FDA-approved drugs and could cause dangerous drug interactions [59, 60]. There are also nutritional and gut microbiome differences between males and females [61]. These microbiome differences can be caused by various factors, including hormones, diet, drugs, BMI, and colonic transit time [61]. Acetaminophen is an over-the-counter drug known to have different toxicity across individuals, which may be due to microbial metabolites that compete with acetaminophen for liver enzyme binding sites [62]. This competition leads to a higher fraction of acetaminophen transformed into a toxic byproduct resulting in increased hepatotoxicity [62].

In substance use, men in the United States are more likely to smoke cigarettes than women [63]. Incomplete combustion leads to accumulations of carcinogenic compounds, which are inducers of CYP enzymes [64], causing many drug interactions, with some drugs requiring dosage increases due to higher CYP1A2 levels [65]. Among biological females who are heavy cigarette smokers, low-dose oral contraceptives have a much higher chance of adverse arterial effects [66]; therefore, the FDA advised doctors not to prescribe oral contraceptives for females smoking over 15 cigarettes a day [67]. Another example of how behavior can lead to biological consequences is the example of alcohol, which interacts with numerous drugs [68]. Alcohol has a stronger effect in women due to differences in alcohol metabolism, leading to higher amounts of alcohol in the body and a higher risk for severe side effects and adverse drug reactions (49). Women are drinking at increased rates on a population scale, and because of their predisposition to stronger effects of alcohol, they are at higher risk for alcohol-related health problems such as liver disease than men [69, 70].

Before reaching 12–17 years old, males are more likely to experience ADEs than females. After adolescence/puberty however, females consistently report more ADEs than males. [13]. The most marked differences in adverse events reported between females and males is between the ages of 18–44 [13]. In addition, there are major sex differences with respect to aging when menopause is considered. Menopause causes major biological changes in the female body, most markedly loss of regular menses and hormonal changes, and occurs around 51 years of age [71]. After menopause, when hormone levels become more comparable to male hormone levels, females still report an excess of adverse drug events but the margin of events between men and women is smaller. Elderly people are most vulnerable to ADEs compared to other groups [72]. One reason for this is that with increased age there is an increase in polypharmacy, which is taking multiple drugs at once. The more drugs a given patient is taking, the greater risk they have for interactions and general side effects [73]. Women, in addition to the elderly, are also more likely to experience polypharmacy, possibly due to their willingness to seek out medical attention more readily [73]. Other contributing factors to the elderly being more susceptible to ADEs are the changes in pharmacokinetics during aging due to changes in renal function [74] and body composition, leading to smaller volumes of distribution for water-soluble drugs such as digoxin, common heart failure medication [75]. Peak concentration of digoxin is increased from 38 hours in younger subjects to 69 hours in elderly subjects, with clearance also reduced in older subjects [75]. As a result, digoxin has a recommended dose for older adults reduced by 20% [75]. This medication also has an increased risk of mortality in women compared to men, which could be due to an interaction between hormone-replacement therapy and digoxin [76].

There are also differences in drug metabolism based on race and ethnicity. In this review, we look at sex as a binary, whereas race is less readily simplified. Race is a social construct that has historically been used to group together people based on outward characteristics such as skin color, presumably based on biological and/or genetic differences. This is not an absolute classification with clear boundaries, with 85% of genetic variation being found within populations and only 15% of genetic variation found between populations [77]. Ethnicity is generally considered to consist of a combination of someone's cultural, religious, or national identity, and is highly subjective. Biological differences in a given population can vary greatly by geographical regions, and are largely affected by socio-economic status [78]. Meaningful biological differences such as an increased rate of heart disease in African Americans can be attributed to decreased access to preventative healthcare, increased concentration of fast-food restaurants and rates of environmental pollution in primarily minority neighborhoods, and more occupational hazards as opposed to genetics [78]. A person with limited access to medical care may under report adverse side effects, or only pursue medical care when side effects are more severe. Racial and ethnic disparities in ADEs have been found in various studies, but consistent definitions of race and ethnicity and evaluation of underlying factors (i.e., environmental and cultural) are lacking [79]. A major study by Man et al. was able to find genetic differences in drug metabolizing and transporter (DMET) allele variants in three different populations: Caucasian, African, and East Asian [80]. In another study, cisplatin, an anti-cancer drug, is more likely to cause nephrotoxicity in African Americans than Caucasians [81]. When intersecting with gender, drug transporter genes ATP7B and KCNJ8 have been shown to have higher mRNA expression in African American women compared to European American women [82]. In the same study, researchers found that there is a significant difference in SLC31A2 in European American males compared to European American females, but not between African American males and females [82]. Race and ethnicity are contributing factors in drug response and ADE outcomes, and should be considered for future studies, especially with sex as a biological variable included.

Due to the overwhelming evidence that males and females have differing responses to many drugs, their treatment recommendations should reflect these discrepancies. Therefore, the need to develop alternative drug treatments to minimize sex-bias related adverse side effects is a high priority, and drug repurposing can help address this in a more timely and cost-effective manner.

### Overview of drug repurposing

As drug discovery costs increase (145% between 2003 and 2013), the need to use systematic methods to identify drug repurposing candidates has grown [83, 84]. Some of these approaches are experimental while others are computational. Experimental approaches conduct drug screens using in vitro and in vivo models testing hundreds to thousands of compounds and evaluating if those compounds affect a specific molecular target or cellular phenotype [1]. An example of a large-scale application of a drug screen is the Profiling Relative Inhibition Simultaneously in Mixtures (PRISM) project [85]. This project treated 930 cancer cell lines with 21,000 drugs to identify which inhibit cancer growth [85]. This large-scale screening process requires many resources including cell lines, drugs, and personnel time and expertise. However, researchers can reduce this investment by identifying specific drug candidates via computational approaches that will prioritize candidates for experimental application.

Over the last several decades, increased processing power and optimized algorithms for rapid calculations have resulted in in silico drug repurposing methods being quicker at identifying drug repurposing candidates than exhaustive experimental approaches (Table 1) [1]. Additionally, the number and size of biomedical databases with appropriate clinical, genomic, transcriptomic, epigenetic, metabolic, and proteomic information for various diseases and preclinical models have expanded [86–88]. These public databases have expedited the process of identifying new drug candidates by making this data accessible to research groups across the world to train their drug repurposing approaches [86–88]. Here we discuss data mining, ligand-target binding prediction, molecular associations, and network computational drug repurposing categories that contain different strategies for identifying drug repurposing candidates.

**Table 1:** Drug repurposing methods overview

Method	Description	Advantages	Disadvantages	Examples
Data Mining	Analysis of data from various sources (including peer-reviewed published experimental data, databases, screens, pharmaceutical information, EHR’s, etc.)	- Crowdsource data - Multiomic data accessible - Reuse of previously analyzed data	- Limited data for rare diseases and understudied drugs, and dependent on large sample sizes - Inconsistency of data structure - Ethics/privacy (for EHR data)	- Mastermind [89] - Pharos [90] - Iwata H et al. 2015 [91] - Duffy Á et al. 2020 [16]
Ligand-Binding Prediction	Interactions between ligands and targets are predicted to determine suitable candidates through binding by structural and chemical simulation	- Identify novel drug targets - Identify novel compound structures - Prior knowledge of protein function not required - Detect possible side effects by off-target binding	- Requires target’s tertiary structure - Experimental binding affinities often not recapitulated - Disregards downstream effects - Computationally expensive - Missing biological context to allow tissue or sex-specificity	- Chupakhin V et al. 2013 [92] - Napolitano F et al. 2013 [93] - Vilar S et al. 2014 [94] - Cao R et al. 2014 [95] - Cheng F et al. 2013 [96]
Molecular Associations	Molecular perturbations are associated with disease, therapeutic outcomes, or drug candidates	- Elucidate drug/disease mechanisms - Compatible with multiomic data - Detect druggable pathways - Exposes off-target drug effects	- High signal-to-noise ratio inhibits deconvolution of signatures - Disregards physiological interactions - Associations may not convey direct causations	- Dr. Insight [97] - signatureSearch [98] - Sanseau P et al. 2012 [99] - Grover MP et al. 2015 [100]
Networks	The relationship of genes within and between pathways provide insight for upstream and downstream drug targets that may infer treatment for a disease phenotype and/or show drug interactions within a biological system	- Multiomic data - Reveals relationships - Determine mechanistic pathways - Exposes off-target drug effects	- Statistically complex - Computationally expensive - Requires strong signal-to-noise or large datasets to deconvolute signal	- Drug2Ways [101] - Green CS et al. 2015 [102]
Experimental—Perturbation Screens	Cultured cells are treated with a variety of drugs and screened for phenotypic response	- Shows gene expression as a result of perturbation - Displays consociation between cell receptors and pharmaceuticals - Non-predicted, in-vitro results	- Immortalized cells - Lacks heterogeneity - Limited microenvironment - Costly	- LINCS L1000 profiles [103] - Iljin K et al. 2009 [104] - Shen M et al. 2018 [105]
Experimental—Binding Assays	The chemical engagement of targets and ligands are tested in vitro to divulge repurposed candidates based on disease-target matching via affinity/thermal stabilization and structures	- Physically measured drug-target binding activity - Captures biophysical features - Reveals promiscuous drug-target interactions	- Disregards downstream effects - Selection of drugs and targets are much more restricted than in silico approaches due to feasibility (cost, time, and accessibility)	- Cellular ThermoStability Assay (CETSA) [106] - Miettinen TP et al. 2014 [107]
Experimental—Animal Models	Organisms are treated with drugs to model patient response and patient-specific disease-causing genetic variants can be introduced to provide more pertinent system	- Recapitulates full physiological system - Resource for multiomic data collection - In-vivo results - Patient-specific models allow for precision medicine	- Significant financial and time expense - Requires narrowed-down list of candidates - Results frequently do not translate to patient response - Orthologous targets may vary greatly from human target structure	- UAB C-PAM [108] - JAX Center for Precision Genetics [109] - BCM Center for Precision Medicine Models [110] - vivoChip [111] - The Hollow Fiber Model [112]

This table describes the methods of drug repurposing with advantages and disadvantages for each. Examples listed were methods used in studies or by consortiums and research centers

Data mining drug repurposing approaches retrospectively analyze information from and across clinical trials, biomedical literature, and other resources with drug outcome or drug target information to identify novel drug indications [113]. These approaches apply machine learning models that use logical and mathematical algorithms to interpret or make predictions about data. For example, Kuenzi et al. generated an interpretable visible neural network, a machine learning model, to predict the effectiveness of drugs for individual cancer mutation profiles [114]. Another example of a data mining approach, text mining, uses biomedical literature to connect information from different studies or data sources to discover novel connections or patterns [115]. For example, aspirin, an over-the-counter medication used for analgesia, was repurposed in 2016 to reduce the risk of developing colorectal cancer after a systematic review of data from the literature and clinical trials [116]. An advantage of these data mining approaches is using large amounts of publicly available data that researchers do not need to recreate for themselves. However, there are some ethical considerations with data mining methods, including data storage, distribution (data should be secure for identifiable information and available for research reproducibility and reuse), and bias within the data, such as the exclusion of different sexes (as discussed in this review), age/developmental groups, and ethnic/racial groups [117]. For example, The Cancer Genome Atlas (TCGA) includes various “omics'' data from tumor and normal samples across several cancers; however, the majority of the samples in this database are caucasian [118]. To overcome this limitation, Gao and Cui applied a machine learning method called transfer learning (this method applies knowledge learned from a large dataset, like TCGA caucasian samples, to a smaller dataset, such as the underrepresented ethnic groups in TCGA) to create ethnicity-specific cancer survival prediction models [118]. This method created a more accurate model than using the limited and underrepresented ethnic samples alone [118]. Furthermore, another limitation of data mining approaches is the dependency on information from literature and clinical trials. If a disease or drug is rare or understudied, there may be limited publications for these approaches.

Additionally, researchers should be aware of data mining challenges with regards to variation in data structure and nomenclature. While the NIH is implementing a new Policy for Data Management and Sharing (DMS Policy) effective January 2023 for NIH-funded research to ensure stricter standards for data sharing and the availability of raw data, this has been a pervasive issue from past data sharing up until this point [119]. Due to different levels of processed vs raw collected processing of data being shared, accompanying metadata being missing or incomplete, and unavailability of code used to manipulate and analyze data, NIH policies are raising the standard to ensure data analyses are reproducible and allow for more effective data reuse through proper data repository use, requiring common format by datatype to maintain consistency, and mandating that full datasets and accompanying metadata be available to the community with “broadest possible terms of reuse” [120]. Additionally, nomenclature for gene names (i.e., Entrez, GenBank, RefSeq, etc.) has remained an issue for data mining, where many synonymous terms and annotations must be searched for in text mining and identification conversion steps for analysis may lead to data loss, errors, or duplication [121]. Ontologies, also known as vocabularies or terminology systems, are also relied heavily upon for literature mining and semantic tools, but introduce inconsistencies and can hinder interoperability [122, 123]. While there have been many attempts to coalesce ontologies, such as Web Ontology Language (OWL), the Open Biomedical Ontologies (OBO) Foundry initiative, and Unified Medical Language System (UMLS) (http://www.nlm.nih.gov/research/umls/), ontology sources often remain incongruent and may lead to misconceptions and error [122]. In summary, there are many factors to consider with data mining approaches, and the impact of these factors should be reduced and limited in order to develop accurate data mining models for drug repurposing.

Another drug repurposing approach, ligand-target binding prediction, identifies drugs predicted to bind to a disease target (i.e., proteins) based on their binding affinity [124]. Molecular dynamic modeling and structure similarity are two types of ligand-target binding prediction methods. Molecular docking predicts if a ligand and a drug target can bind via their structures [124]. A limitation of this approach is that it requires a significant amount of time and memory, even on a high-performance computing system [125]. Some molecular docking methods have reduced the complexity of calculations by approximating and removing certain parameters to increase computational speeds, but this has increased docking energy errors and unreliable ranks of drug candidates [126]. Alternatively, structure similarity approaches predict drug candidates based on the premise that similar drugs will have similar mechanisms of action or adverse events [127]. Ligand-target binding predictions are limited due to the requirement for accurate information about drug structures, mechanism of actions, and protein structures of disease targets to predict suitable drug candidates, which are often inaccurate or unknown [1].

Molecular association methods identify targets or patterns from molecular data (i.e., genomic, transcriptomic, epigenetic, metabolic, or proteomic profiles) that correlate with disease, therapeutic outcomes, and/or drug candidates [1]. Molecular association strategies include guilt-by-association, signature matching, and signature reversion [1]. Recently, a genome-wide association study (GWAS) used guilt-by-association to identify drug repurposing candidates for psoriasis [128]. In this study, IL-23 receptor gene variants were found to be associated with the development of psoriasis, and therefore the IL-23 receptor became a potential drug target [128]. Further, based on biomedical literature, risankizumab was identified as a drug candidate because it targets the IL-23 receptor, and after clinical trials, risankizumab was indeed approved for psoriasis treatment [128, 129]. Signature matching, another molecular association strategy, has been applied in several cancer applications, as reviewed in Wang et al. [129]. It compares patient molecular profiles to cell line profiles or another model system that were treated with drugs and assessed for a specific phenotype (e.g.., cell viability in cancer cell lines) [129]. Another variation, signature reversion, leverages molecular disease signatures (i.e., gene expression differences between disease and normal) and cell line perturbation signatures (i.e., gene expression differences before and after drug treatment) to identify drug signatures that are inversely related to disease signatures [88]. Chen et al. applied this principle to liver cancer and identified and validated four drug repurposing candidates in xenograft mouse models [130]. Signature matching and reversion can be approached by either enrichment statistics such as Kolmogorov–Smirnov or correlation methods [131]. However, enrichment statistics approaches had lower accuracy compared to correlation approaches, but correlation approaches were more sensitive to noise [98, 131]. Additionally, molecular associations methods can only determine correlation and not causation; therefore, molecular associations are not always the drug target or the cause of the disease. For example, if a GWAS study identified a gene variant with favored drug response, it should not conclude that the gene with the variant or gene closest to the variant is the drug target. A neighboring gene could be the drug target due to the influence of linkage disequilibrium where genes near each other tend to be inherited together [1]. Therefore, researchers using these molecular association strategies should be critical when evaluating and interpreting their associations to avoid making causal inferences about drug targets [1].

Network approaches evaluate mathematical graphs (nodes joined together by edges) representing relationships as edges between different nodes like genes, proteins, diseases, and drugs to identify drug repurposing candidates [132]. One of the benefits of this approach is that networks can integrate multiple data types to predict drug candidates. In one study, Morselli et al. successfully repurposed four drug repurposing candidates for COVID-19 by implementing a protein–protein interaction network, information about severe acute respiratory syndrome coronavirus 2 (SARS-CoV-2), and drug targets [133]. Another benefit of a network approach is interpretability. This interpretability allows for insights into possible disease and drug mechanisms. For example, a study that used tissue-specific networks derived from transcription factor sequence motifs, protein–protein interaction, and gene expression data identified the BTK inhibitor ibrutinib as a drug candidate for metabolic syndrome [134]. Because of the interpretability of their network approach, the researchers gained mechanistic insight into how ibrutinib treatment might treat metabolic syndrome via BTK expression and immune cells [134]. However, some drug-target interaction networks with nodes representing drugs and gene targets have a high number of false positives due to nonspecific drug targets making it difficult for this approach to predict new drug candidates [135]. A prime example of one multitarget drug is imatinib, which was originally designed for its inhibition of BCR-Abl fusion protein, but was also found to be especially efficacious in chronic myeloid leukemia by also inhibiting non-oncogenic c-Abl tyrosine kinase in normal cells [136]. Another limitation is the dynamic nature of biological systems means networks capture a specific point in time so critical evaluation and interpretation of network construction is necessary [135]. Additionally, network analysis can require costly computing resources and time due to complex algorithms [137].

However, a promising approach is to use a combination of computational and experimental approaches to identify and validate drug repurposing candidates (Table 1). For example, Fang, et al. conducted a study for Alzheimer’s Disease (AD) with data mining approaches and a drug-target network to identify a drug repurposing candidate, sildenafil, followed by experimental assays to validate its mechanism of action in patient-derived induced pluripotent stem cells (iPSCs) [138]. This study used multiple data sources including health insurance claims from the MarketScan Medicare Claims database and gene expression data from the Gene Expression Omnibus (GEO) and Genotype-Tissue Expression (GTEx) databases [138]. Moreover, some studies combine several methods (known as weak learners) into a single framework, resulting in better therapeutic predictions than using one method alone [131]. This technique is called an ensemble approach. EMUDRA, an example of an ensemble method, combined four weak learners: Kolmogorov–Smirnov statistic, weighted signed statistic, the sum of fold changes, and cosine similarity [131]. This ensemble model outperformed the individual weak learners and other drug repurposing approaches with simulated and drug perturbation data [131]. This methodology performs better in cases where the weak learners have similar accuracy but diverse predictions [139, 140]. In this situation, the different weak learners' algorithms identify different important signals to determine drug candidates. A limitation of this approach is the increase in computational complexity, which requires more computational power and time to predict drug repurposing candidates.

Lastly, with some complex diseases such as cancer, the use of combinational drug therapy increases the rate of success because different combinations of drugs can have synergistic effects on the same target or multiple targets [141]. For example, multiple drugs can be used to synergistically impact one target or pathway, such as GKT136901 and L-NAME working on NOX4 and co-target NOS [142]. Several computational methods are available to investigate synergistic effects between drugs for therapy [143, 144, 145, 146]. In a computational development challenge to find cancer drug combinations, 160 teams developed computational methods to find synergistic drug combinations [146]. After reviewing the performance of the methods developed by the different teams, this project concluded that ensemble approaches with multiple computational methods improved drug combination predictions compared to single methods [146]. Similar to the ensemble approach, combinational drug repurposing methods increase the complexity of the model, affecting computational power and time.

In this section and Table 1, we highlighted several computational methods that have prioritized novel drug repurposing candidates and their limitations which can significantly impact the success or accuracy of drug repurposing. Overall, critical evaluation of current and future computational methods via code peer-review and in silico and experimental validation is important to continually improve computational drug repurposing [147]. In addition, many ethical considerations that should be acknowledged when developing or using computational models because all models are designed with different assumptions and biases due to algorithms or datasets used to train models [148]. Understanding the limitations of a model will help identify if the method is suitable for the task or if another model with less bias or better assumptions should be used or designed [148]. Another common hurdle for all of these methods is the requirement for statistically powered datasets to create more accurate models. This limitation can be incredibly challenging for sex-aware drug repurposing because these methods require powered datasets for males and females. In combination with other challenges to studying sex differences (discussed in the last section), these limitations make sex-aware drug repurposing difficult. Still, we propose and discuss several solutions and drug repurposing approaches to mitigate these challenges to aid in the development of better sex-aware drug repurposing approaches (discussed in the next section).

### Sex-aware drug repurposing methods

Currently, there are limited methods available to either select sex-specific drug repurposing candidates that will be effective against a disease of interest or prioritize candidates to avoid SBAEs. While these methods fall under the same drug repurposing categories, the following sex-aware methods are variations that adjusts input data, parameters, and/or algorithms for sex differences to identify sex-specific drug candidates or SBAEs. We did not consider drug repurposing methods that used sex as a covariate as sex-aware because using sex as a covariate reduces the impact of sex in computational models. These models will not identify sex-specific drug candidates and adverse events. Here we summarize the currently available sex-aware approaches or studies for sex-aware drug repurposing (Table 2).

**Table 2:** Sex-aware drug repurposing examples

Method	Examples	Development	Sex-Aware Approach
Data Mining	Drug Central [149]	Database	Drug Database compilation using FDA, EMA, and PMDA; information includes active ingredients, MOA’s, indivations, pharmacological actions, regulatory data, chemical structure, and adverse drug events separated by sex to help correct for sex-bias
	AwareDX [7]	Study/Analysis	Pharmacovigilance algorithm that predicts sex-bias adverse events from FAERS data and found 20,817 sex-specific drug risks
	“Sex differences in pharmacokinetics predict adverse drug reactions in women” ([14])	Study/Analysis	Pharmacokinetic differences by sex are linked to sex-specific adverse drug reactions using data procured from ISI Web of Science and PubMed
Molecular Association	“Gender differences in the effects of cardiovascular drugs” [18]	Study/Analysis	Sex influences on pharmacokinetics, pharmacodynamics, and other physiological factors are reviewed for cardiovascular drug response
	“Brd4-bound enhancers drive cell-intrinsic sex differences in glioblastoma” [150]	Study/Analysis	Sex-specific epigenetic signatures are identified in GBM mouse astrocytes and human glioblastoma stem cells
	“Sex-Dependent Gene Co-Expression in the Human Body” [25]	Study/Analysis	Across-tissue RNAseq analysis finds co-expression to be highly sex-dependent
Networks	“Population-scale identification of differential adverse events before and during a pandemic” [9]	Study/Analysis	Sex-specific desparities are presented in network analysis of adverse drug events before and during COVID-19 pandemic
	“Gene regulatory network analysis identifies sex-linked differences in colon cancer drug metabolism” [17]	Analysis using PANDA and LIONESS	Molecular differences investigated using sex-specific networks to uncover role in metabolism of drugs in colon cancer
	“Sex Differences in Gene Expression and Regulatory Networks across 29 Human Tissues” [151]	Analysis using LIONESS	Sex biases are found in patient-specific networks in every tissue and by disease
	“Detecting phenotype-driven transitions in regulatory network structure” [152]	Analysis using ALPACA	Sexual dimorphism are investigated in human breast tissue gene expression networks
Ligand-Binding Prediction	“3D pharmacophoric similarity improves multi adverse drug event identification in pharmacovigilance” [165]	Study/Analysis	Pharmaceutical 3D structure similarity predictions are combined with adverse drug events as a method that may be applied for comparing safety by sex-aware reporting
Experimental	“Sexual differentiation of central vasopressin and vasotocin systems in vertebrates: different mechanisms, similar endpoints” [153]	Study/Analysis	Rat model is used in comparison with human model to compare sex-bias of common neuropsychiatric drug targets

Studies, tools, and databases that have taken sex into account for drug repurposing are described here in this table. The main method is listed (as described in Table 1) as well as examples and a short explanation of how the method integrated sex-specific awareness

The first sex-aware drug repurposing approaches use data mining to identify SBAEs based on case information from patient adverse events databases such as FAERS or VigiBase. A study from Yu et al. calculated the reporting odds ratio for a sex-bias adverse event based on FAERS’ case reports [11]. They found and confirmed several SBAEs and drug combinations via drug labels or previous studies [11]. The drug repurposing database DrugCentral also used the FAERS database to calculate the likelihood ratio for a sex-bias adverse event for all drugs in the database. These calculations can identify drugs with SBAEs and prioritize drug candidates that avoid SBAEs [149]. Another study created a random forest model based on the FAERS database to predict a propensity score (the likelihood that a patient is female) based on clinical data and the standardized medical terminology used for medical conditions, medicines, and medical devices (Medical Dictionary for Regulatory Activities MedDRA) adverse events terms [7]. This study used several metrics such as out-of-bag score and Receiver Operating Characteristic Area Under the Curve (ROC-AUC) to evaluate their random forest model. This model had an out-of-bag score of 0.63 and ROC-AUC of 0.64 [7]. However, this model had a low recall of 0.47 and required 250 patients per sex for each adverse event [7]. A more recent study from Zucker and Prendergast conducted a literature search to identify SABEs based on pharmacokinetic differences between males and females [14]. This approach was successful in identifying female-bias adverse events but struggled to identify male-bias adverse events [14]. This limitation might suggest pharmacokinetics are less likely to cause male-biased adverse events [14]. In addition, this study was limited by only having pharmacokinetic information for a small fraction of FDA-approved drugs [14].

Furthermore, there are some additional limitations with data mining studies. For example, adverse events are often voluntarily reported by healthcare professionals, consumers, and drug manufacturers [154]. Even with standardized terminology like MedDRA, it can be difficult to categorize or describe an adverse event; therefore, there could be misclassification of a specific adverse event. Second, case reports cannot distinguish an adverse event caused by a drug or an extraneous factor such as another drug. Some databases like FAERS do not require a causal relationship for a report to be filed [154]. Therefore, it is difficult to determine if a drug causes an adverse event. However, it is possible to estimate disproportionality or calculate odds ratios to identify associations between drugs and adverse events [9].

Next, we identified two sex-aware molecular association studies that used molecular biomarkers to identify sex-specific drug candidates or SBAEs. The first, a study by Kfoury et al., identified drug repositioning candidates not currently FDA approved for any condition that might benefit GBM patients [3]. This group previously reported that GBM is sexually dimorphic because of the variation in gene expression profiles between males and females that they associated with different survival between the sexes [3]. After this study, they hypothesized that the gene expression variation might be due to sex differences in epigenetic regulation [3]. Specifically, Kfoury et al. investigated the bromodomain and extraterminal (BET) family of proteins, epigenetic readers of histone lysine acetylation [150]. Their study identified that BET inhibitors (JQ1 and RVX208) decreased tumor growth in male tumors but increased the growth in female tumors [150]. This study exemplifies how molecular differences between males and females can lead to a hypothesis resulting in the discovery of a drug candidate. Another study analyzed drug perturbation profiles from Connectivity MAP, an extensive drug perturbation by cell line database, to determine what drugs perturbed heart-specific sex-biased genes as determined by differential gene expression analysis [155]. With this information about which drugs perturbed heart-specific sex-bias genes, they found sex-bias drug responses for acebutolol, tacrine, and metformin in rat models and further validated their results with clinical information from a human patient cohort [155]. Currently, this sex- and tissue-specific approach is limited to heart tissue because they only investigated sex-biased gene expression in heart tissue. However, researchers can adapt this method to develop more tissue-specific and sex-aware models.

One sex-aware experimental approach is the manipulation of sex hormones as a therapy for a disease. Such manipulation is used when one sex tends to respond better to current therapies and/or have better outcomes than the other. For example, in AD, which is more prevalent in females compared to males, leuprolide acetate, an androgen deprivation therapy used for the treatment of prostate cancer, has been investigated for the treatment of AD as it might slow the progression of the disease [156, 157]. This approach requires that the sex differences in disease outcome is due to hormone differences and not other factors (i.e., genetic or epigenetic differences) that can cause sex differences in disease outcome or drug response.

While most of these methods performed poorly due to limited, sex-balanced datasets. Future development of strategies to more accurately model sex-bias from these unbalanced datasets via methods similar to transfer learning approaches done by Gao and Cui study or leverage new and more balanced datasets is required. Furthermore, sex impacts biological systems in multiple ways (i.e., genetics, epigenetics, etc.) [20, 33]. This means that computational models could be over-simplified and inaccurate by treating sex as a single biological variable instead of a factor influencing several biological variables in a drug repurposing model [158]. In addition, the influence of sex being understudied means that the true complexity of sex is unknown for developing or adjusting drug repurposing methods and challenging for interpretation of the drug repurposing candidates [33]. This is especially important to consider when sex differences can vary across different tissues, ethnic groups, age and development groups, diseases, and perturbations [22, 33]. It is also surprising given genomics being a common data source for drug repurposing methods and sex determination strongly influenced by sex chromosomes that there are few available methods that use genomic data to predict sex-aware drug repurposing candidates and SBAEs besides the Cui et al. study for heart and sex -specific drug repurposing candidates [155]. Also, we were unable to identify sex-aware methods for network or ligand-binding drug repurposing categories. Therefore, we suggest approaches to improve sex-aware drug repurposing for data mining, ligand-target binding prediction, molecular associations, and network computational drug repurposing categories.

Molecular association and network approaches are promising candidates for future sex-aware drug repurposing methodologies. Molecular association studies provide molecular biomarkers that might be causal for different responses to drugs [155]. These methods can separate males and females to identify genes or other biomarkers to determine drug repurposing candidates or prioritize drugs. The GTEx study discovered that gene expression differences between males and females tend to be small [22]. An alternate approach to evaluating gene-level sex differences or individual biomarkers would be to develop metagenes, signatures, or low-dimensional representations of gene expression, DNA variation, or protein expression to identify drug repurposing candidates. Using low-dimensional representations of molecular patterns reduces the multiple hypothesis testing burden to find significant differences between males and females [159]. These differences could be associated with drug response or adverse events.

As an emerging computational approach for sex-aware drug repurposing, network techniques developed by the Network Zoo have been used to build gene regulatory networks to identify regulatory pattern differences between males and females from GTEx tissue samples [151]. The authors observed larger sex differences between the edges in the gene regulatory networks than gene expression [151]. They also showed sex differences in the regulatory pattern of drug metabolism in colon cancer, indicating a possible sex difference in drug response [17]. Another network method developed by the Network Zoo group, ALPACA (ALtered Partitions Across Community Architectures), determined network module sex differences in breast tissues [152]. These differences were associated with intracellular estrogen receptor signaling pathways, developmental and signaling pathways, and pathways related to breast cancer [152]. While these network approaches did not identify candidates for drug repurposing, sex-specific networks capture variation due to sex differences better than differential gene expression because network approaches identified more significant differences between edges and network communities (i.e., groups of related nodes and edges in networks) in male and female gene regulatory networks compared to differences in gene expression [151, 152]. Therefore, the use of male and female networks and current network methods is a potential future direction.

Due to several limitations, data mining and ligand-target binding prediction approaches are challenging to adapt for sex-aware drug repurposing. First, data mining approaches typically require large amounts of balanced data, equal male and female data points. However, several databases are not balanced. For example, the UK biobank is more female-biased (as of 2021: ~ 273,000 females and males 229,00) while GTEx is more male-biased (v8 release: 636 males and 312 females) [22, 160, 161]. In addition, retroactive studies should consider using downsampling techniques because older clinical trials did not require female subjects, biasing clinical studies toward male subjects [20]. For basic biomedical research data, human samples are slightly female-biased (52.1%) while mice samples are male-biased (62.5%) [162]. Furthermore, this bias varies between different biomedical research disciplines with reproductive studies having more female-only studies while pharmacology has more male-specific studies [163]. Also, studies have historically failed to report the sex for their samples [162]. Therefore, future studies should carefully consider what datasets are being used and apply methods to overcome disproportional datasets to create sex-aware data mining approaches.

Ligand-target binding prediction methods could also be difficult to adjust for sex differences. Several studies have identified that sex differences can be due to gene regulatory and hormone signaling [22, 151]. Therefore, ligand-target binding prediction, a method that only evaluates how a ligand and target interacts, does not traditionally consider how other influences such as hormones will affect the ligand-target binding interaction [164, 151]. This is also a limitation for experimental target binding assays, too. However, one potential avenue is to compare structures of drugs with known sex-bias responses to identify drugs with potential for sex-bias drug responses. This sex-aware approach is similar to Vilar et al.’s approach, which compares drugs’ structures with known adverse events to identify drugs with potential for adverse events [165]. For molecular docking, future researchers should consider the expression of drug targets between the sexes. For example, if a target is highly expressed in one sex but not the other, a drug candidate from molecular docking methods might be only therapeutic in one sex. Also, ligand-target binding prediction methods should evaluate if the drug target might be in sex-bias sub-networks, influenced by sex-bias transcription factors, or regulated by sex hormones. Ideally, the development of ligand-target methods that considers all of these factors before predicting candidates would be the most useful tool for sex-aware drug repurposing. Overall, the current methods are not sufficiently developed for sex-aware drug repurposing, but they have the potential.

### Challenges and proposed solutions for using model systems for sex-aware drug repurposing

**Fig. 2:**
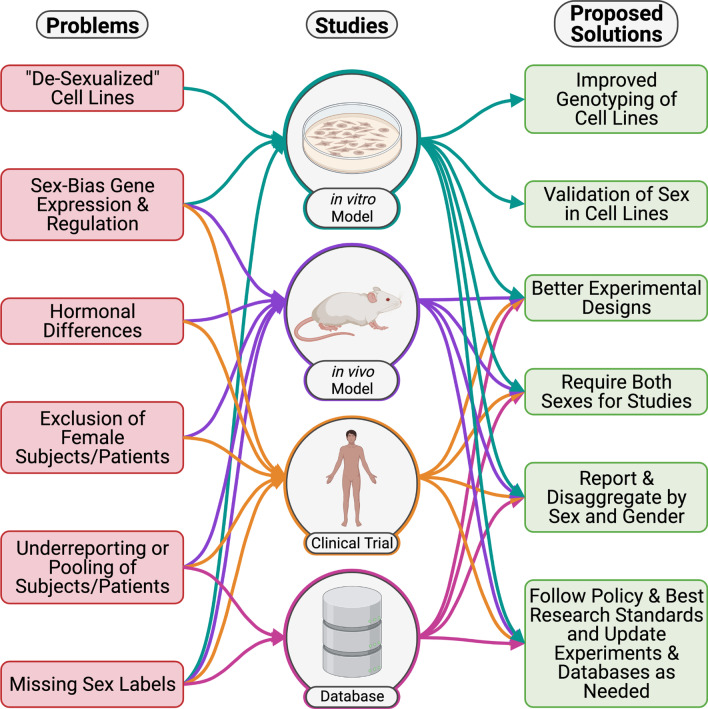
Proposed solutions to sex-aware drug repurposing challenges. Teal arrows are connected to cell lines models. Purple arrows are connected to preclinical models. Orange arrows are connected to clinical trials. Pink arrows are connected to databases

Several challenges exist across basic, translational, and clinical research in assessing sex as a biological variable (SABV) in in vitro, in vivo, and in silico model systems (Fig. 2). These challenges impact sex-aware drug repurposing because these models are critical for identifying and validating drug repurposing candidates. Here we discuss the challenges and proposed solutions for these model systems.

The genomic basis of sexual differentiation is a confounding factor for all in vitro, in vivo, and in silico model systems. Sex chromosomes experience lower accuracy than autosomes for genotype arrays because of the homologous regions between the X and Y chromosomes [33]. Many previous GWAS studies that determined SNPs from genotyping assays removed sex chromosomes from their analysis [166]. This resulted in an underrepresentation of SNPs from sex chromosome regions in analyses [166]. Therefore, GWAS studies including sex chromsome SNPS due to improved genotyping arrays and analyses are needed to determine the influence of sex chromosomes. Additionally, several studies have also demonstrated poor mapping quality of sequencing data and bias from homologous regions between the sex chromosomes, reducing the ability to detect sex chromosome DNA variation and gene expression accurately [33]. However, approaches are now being used to remedy these issues. For example, XYalign, a bioinformatics framework, can be applied to next-generation sequencing data to appropriately account for the sequence homology between the X and Y chromosome by inferring the sex chromosome ploidy of a sample and remapping the sequencing reads to the sex complement of the sample [167]. Another approach creates sex-specific reference genomes for sequencing read alignment [168]. This approach has been shown to result in more accurate read alignment for both traditional aligners (i.e., STAR, HISAT) and pseudo-aligners (i.e., Salmon) [168]. Correct alignment is important for DNA variants on the sex chromosomes because, for example, the *CTPS2* and *DLG3* X chromosome genes are known to cause differences in drug response ([169]. Further, the expression of these genes are correlated to the sensitivity of both platinating agents carboplatin and cisplatin [169].

In vitro cell line models are important to drug repurposing because many drug screens and validation experiments use these models for testing the efficacy and toxicity of drug repurposing candidates. However, some cell lines become “de-sexualized” after losing a Y chromosome, in the case of male cell lines, or the loss of an X chromosome, in the case of female cell lines [170]. Additionally, after several passages (the number of times a cell line cultured has been harvested and reseeded), female iPSCs will frequently undergo inactive X erosion from loss of *XIST* expression (the long coding RNA that causes X inactivation) and reactivate expression of silenced genes on the inactive X chromosome, a process known as inactive X erosion [171]. Researchers can validate the sex of in vitro models to make their results more rigorous by considering SABV in cell lines. Overall, this will improve experimental drug repurposing screens' ability to predict and validate effective and safe candidates.

Additionally, genetic and hormonal sex differences are also difficult to account for in vivo animal models as these can vary across organisms [172]. While there are several benefits to in vivo models such as the ability to test hypotheses in dynamic biological systems, animal models are still not perfect mimics of human biology. For instance, most animals do not follow the same sex determination as mammals [172]. For example, in the *Drosophila* genus of flies, XX, XXY, and XXYY flies are female; while XY and XO flies are male [173]. In flies, The Y chromosome does not impact sex determination and both X chromosomes remain active [173]. Another widely used model organism, *C. elegans,* has XX hermaphrodites and XO males [173]. In addition, zebrafish have several different loci across the genome that determines sex [174]. Overall, all of these model organisms have been used in drug screens for drug repurposing candidates, but future studies can further investigate how sex difference for model organisms compare to human sex differences [175, 176, 177].

Another challenge for in vivo models is sex differences in human phenotypes might not be present in a given model organism. For example, the longevity between males and females varies significantly between species and human females, similar to yellow baboons, tend to live longer [178]. Mice, a common model organism in biomedical research, also demonstrate variations in longevity [179]. For some mouse strains, the males live longer (ie., 129S1, NOD.B10, and NZW), but in others the females live longer (ie., B10 and P) [179]. However, if mouse strains are pooled together, mice do not show a sex difference in longevity [179]. Another consideration is hormone differences between humans and model organisms. One study compared rats, mice, and humans by measuring sex hormone levels at different points in development [180]. They found that these sex hormones peak at different developmental stages for each organism [180]. Also, rodents have estrous cycles in which the uterine lining is reabsorbed instead of removed, such as in the menstrual cycle in human females [181]. At a later age (around 9–12 months), the estrous cycle becomes irregular and acyclic, similar to human menopause, but there is evidence of mature ovulatory follicles, neo-oogenesis, and no extreme decline of estrogen levels in rodents [181]. The lack of mature follicles and the significant decline of estrogen levels are hallmarks of human menopause [181]. Currently, there are three different rodent models for menopause, including the ovary-intact model to investigate the aging hypothalamic-pituitary–gonadal axis, ovariectomy, and the use of 4-vinylcyclohexene diepoxide, which reduces the fertility in rodents, mimics the transitional menopause in humans [181]. Overall, how well model organisms reflect sex differences in humans across different contexts needs further investigation. For example, a recent study suggested that sex-bias gene expression in the proximal tubule cells of kidney in humans did not match sex-bias gene expression in mouse proximal tubule cells [182]. With the kidney being an important organ for drug metabolism, this finding can have a major implication for the modeling of drug responses in mice.

In vivo animal studies have historically excluded female animals from many study designs, resulting in less female data [183]. For example, in publicly available gene expression data (RNA-Seq and Microarray), 62.5% of labeled mice gene expression samples are male [162]. Stated reasons for excluding female animal models include the perceived need to account for the estrous cycle in female rodents, increase sample sizes of female subjects for statistical power, and increased time and costs associated with these factors [164, 184, 185, 186]. Recently, studies have reported that hormonal fluctuations in animal models do not necessarily lead to increased variability in results for either of the sexes [164, 184–186]. Therefore, well-powered studies can be designed with minimal increase in sample sizes (i.e., 14–33%) that still observe the effects of and interactions between two independent variables [187]. Studies can do this by employing factorial study designs which utilize a 2-way analysis of variance (ANOVA) to discern outcomes due to sex differences from those that result from experimentation [183, 187, 188, 189]. Another approach to further understand the impact of sex chromosomes compared to hormones in mice models is the use of designed studies with models that can discern the effects of sex chromosomes from those of gonadal hormones. For example, hormonal influences can be minimized by using functionally gonadectomized mice, such as the *Sf1* knockout mouse [190, 191], sex hormone receptor knockout mouce [192, 193], or the four-core genotypes model [191, 194, 195]. In this model, the *Sry* gene is moved from the Y chromosome to an autosome to generate four genotypes: XXF (XX mice with ovaries), XXM (XX mice with testes), XYF (XY mice with ovaries), and XYM (XY mice with testes) [191, 194, 195]. Not only does this allow for observation of gonadal hormone effects separately from sex chromosome effects, but it also identifies sex chromosome influences on non-gonadal tissues [191]. In the future, in vivo studies can incorporate other factors such as development/aging and reproductive events (i.e., puberty, pregnancy, and menopause). These events have hormone fluctuations that can also impact sex-bias transcriptomic regulation and drug responses [19, 196].

The biological impact of sex extends also to impacts on in silico modeling of biological systems. The exclusion of female animals in preclinical studies [183] and the low enrollment of female patients in clinical trials [197, 198] have led to a decrease in data for female subjects and underpowered statistical results for retrospective analyses desegregated by sex [183]. There are also the problems of underreporting (not including the distribution of sex across samples) and pooling (acknowledging that both sexes were used in the study but the study did not analyze data for the impact of sex) [184, 199]. With the recent implementation of NIH’s SABV policy in 2016, underreporting of sex has decreased between 2009 and 2019 from 16 to 6% in biomedical research articles [163]. However, sample pooling is still common in studies with both male and female samples (42% in 2019 and 50% in 2009) [163]. Both underreporting and pooling reduce the reproducibility and transparency of scientific research because it masks biological differences between the sexes [184, 199]. This leads to data accuracy issues and misinterpretation of the results from the study [184, 199]. With genomic data, there are ways to infer sex if the study does not report them. Researchers can identify the ploidy of the X chromosome or develop sex marker sequences from sex chromosome nucleotide sequences [167, 200]. Another method developed by Fylnn et al. identified the sex of a sample by the use of an elastic net machine learning classifier [162]. This classifier had an accuracy of 91% in microarray and 88% in RNA-seq human gene expression data [162]. With sufficiently powered data from both male and female subjects across basic, translational, and clinical research, increased quality data will improve in silico models and thus the precision and efficacy of sex-aware drug repurposing approaches.

### Perspectives and significance

We envision sex-aware drug repurposing as a standard analysis used in drug repurposing research due to the overwhelming evidence that sex is important for drug response. Even if a disease does not show a known sex difference, the variation of drug responses between the sexes warrants investigations of SBAEs and drugs that might have sex-specific therapeutic effects. While several drug repurposing strategies attempt to find drug candidates without the influence of sex, sex-aware drug repurposing identifies drug candidates that will have differential effects between the sexes by either having variations in therapeutic effects between the sexes or cause an adverse event in one sex (i.e., SBAEs). In this review, we highlight several FDA-approved drugs and drug candidates that have different therapeutic effects, such as the BET inhibitor drug candidate for GBM [150]. A potential impact of sex-aware drug repurposing (and sex-aware drug discovery not discussed in this review) would be more drugs being approved for only one sex for non-sex-specific conditions due to the difference in therapeutic effectiveness or to avoid an adverse event. Several drugs are only FDA-approved for a condition that occurs in one sex (i.e., ovarian or testicular cancer), but in 2019, an HIV prevention medication, Descovy, was approved for cisgender men and transgender women due to a large clinical trial with just cisgender men and transgender women [201]. While there is justified criticism for the approval of a drug for a specific gender/sex due to the underrepresentation of females in a clinical trial, this case highlights that the FDA can approve drugs in a sex and gender-specific manner even though the condition is not sex or gender-specific [201]. Another future consequence of sex-aware drug repurposing is the practice of adjusting dosage based on sex-specific pharmacokinetic or pharmacodynamics, as suggested for the FDA Ambien example discussed earlier [51]. We believe as sex-aware drug repurposing expands and develops that (1) there should be standards or guidelines for doctors to aid in differentiating prescriptions between the sexes, (2) drug manufacturers should inform clinicians about sex differences on dosage, efficacious, and side effects, and (3) government agencies like the FDA should require SBAEs and sex-specific dosage be on labels and information packets for patients. Currently, many government agencies are encouraging these changes to happen [202, 203]. Finally, we hypothesize that the development of sex-aware drug repurposing methods is the first step in improving drug repurposing and drug discovery methods.

## Conclusion

Here we described sex-aware drug repurposing and discussed the challenges and future of sex-aware drug repurposing. Drug repurposing is a valuable method for identifying drug candidates for FDA approval because of its ability to prioritize efficacious drug candidates at a reduced cost compared to traditional drug discovery [1, 83]. However, various drugs have male and female-bias responses and adverse events [14, 49]. This variation in drug response arises because of various sex differences in genetic, epigenetic, hormonal, immunological, metabolic, and environmental factors [33]. Several computational drug repurposing approaches exist or are being developed to identify or prioritize drug candidates for both sexes [7, 11, 17, 25, 149, 150, 152, 155]. This can lead to improved therapeutic options and prevent adverse events for patients. In addition, these drug candidates could provide novel insights into disease manifestation, progression, and underlying mechanisms. This can be beneficial to understanding and treating diseases, such as in the case of the BET inhibitor for GBM discussed [150]. Unfortunately, the validation of these drug candidates is limited by existing preclinical models [162, 184]. Therefore, in line with NIH policy, future studies should routinely investigate how including sex as a biological variable influences study design.

There is an urgent need to address the following: (1) the lack of balanced data to develop accurate models for sex-aware drug repurposing, (2) the need for a variety of improved sex-aware drug repurposing methods, and (3) the scarcity of studies relating to sex differences and variation in drug response between the sexes. Increased representation of females in biomedical research and clinical trials through balanced sex studies or female-only studies is needed to improve drug repurposing approaches. While some methods are available to overcome limited datasets [7, 11, 17, 25, 149, 150, 152, 155], ultimately statistically powered datasets provide more accurate modeling and predictions. Furthermore, one of the sex-aware drug repurposing methods that investigated pharmacokinetic sex differences (i.e., Zucker and Prendergast) did not find many male-bias adverse events based on pharmacokinetics [14]. We hypothesize that male-bias adverse events due to pharmacokinetics have already been addressed in the early phases of clinical trials due to males being the majority of subjects. However, due to the underrepresentation of female subjects, pharmacokinetic sex differences are not identified in early clinical studies, which might be the reason the Zucker and Prendergast study only identified female-biased adverse events. This highlights the importance of sex-balanced studies and clinical trials. In addition, the current methods are inadequate for exploring sex-aware drug repurposing and have performance limitations due to the data types used. Also, while sex-aware methods for data mining and molecular association approaches have been developed, there is a lack of sex-aware drug repurposing approaches that apply ligand-target binding prediction and network methods. The development of novel approaches is crucial for identifying future drug repurposing candidates for both sexes. In addition, the field needs sex-aware drug repurposing approaches for different omics data such as epigenetics, metabolomics, etc., which have been beneficial in other drug repurposing methods that do not consider sex [87, 114]. The current sex-aware methods are biased towards clinical, genomic, and transcriptomic input data. Lastly, there is a need for more studies that focus on sex differences across all diseases (if the disease occurs in both sexes). Even five years after the US National Institutes of Health required studies to consider sex as a biological variable, there are still many understudied aspects surrounding sex differences and how they affect drug response [163, 204]. The information about sex differences could inspire and improve drug repurposing methods in the future.

Upon reflection, one promising sign that sex-aware methods will improve the field of drug repurposing is how tissue-aware drug repurposing has improved the field. While sex is an essential factor in drug response, other factors such as tissue differences, aging, development, race, social, and environmental factors are also important to consider for drug repurposing methods. For example, a study investigated tissue-specific genetic features of drug target genes (i.e., tissue specificity of gene expression, Mendelian association, phenotype, and tissue-level effects of genome-wide associations loci driven by eQTLs, and genetic constraint) [16]. They discovered that these tissue-specific features resulted in a 2.6 more significant risk of side effects, and drug development and repurposing studies could use these tissue-specific genetic features to help evaluate drugs [16]. Overall, the use of tissue-specific gene expression improved drug safety and efficacy predictions in multiple studies [16, 205]. This indicates that as drug repurposing expands to account for sex differences, drug safety and efficacy predictions will also improve for both sexes.

In conclusion, the development of sex-aware drug repurposing methods is essential but challenging due to the understudied complexity of sex differences. We recognize these challenges for sex-aware drug repurposing, but its potential for biomedical research and patient care outweighs the difficulties. In the future, sex-aware drug repurposing will identify safer and more efficacious drug candidates for both males and females.

## Data Availability

Not applicable.

## Abbreviations

NIH: National Institute of Health
SABV: Sex as a biological variable
SAGER: Sex and gender equity in research
SBAE: Sex-bias adverse event
FDA: Food and Drug Administration
SLE: Systemic lupus erythematosus
GBM: Glioblastoma
COVID-19: Coronavirus disease of 2019
MGMT: O6-methylguanine-DNA methyltransferase
eQTLs: Expression quantitative trait loci
XCI: X-chromosome inactivation
H3K9/14ac: Histone H3 Lys9 acetylation
H3K9me3: Histone H3 Lys9 trimethylation
H3K27me3: Histone H3 Lys27 trimethylation
HDAC: Histone deacetylases
CYP: Cytochrome p450 enzymes
PRISM: Profiling relative inhibition simultaneously in mixtures
GWAS: Genome-wide association study
SARS-CoV-2: Severe acute respiratory syndrome coronavirus 2
ANOVA: Analysis of variance
AD: Alzheimer’s disease
FAERS: Food and Drug Administration Adverse Event Reporting System
ROC-AUC: Receiver operating characteristic area under the curve
MedDRA: Medical Dictionary for Regulatory Activities
GTEx: Genotype-Tissue Expression project
GEO: Gene expression omnibus
BET: Bromodomain and extraterminal proteins
ALPACA: ALtered Partitions Across Community Architectures
iPSCs: Induced pluripotent stem cells
EHR: Electronic health records
EMA: European Medicines Agency
PMDA: Pharmaceuticals and Medical Devices Agency
MOA: Mechanism of action
TCGA: The Cancer Genome Atlas
OWL: Web Ontology Language
OBO: Open Biomedical Ontologies Foundry initiative
UMLS: Unified Medical Language System
